# Bis(4,7-dichloro-1,10-phenanthroline-κ^2^ *N*,*N*′)bis­(dicyanamido-κ*N*)copper(II)

**DOI:** 10.1107/S1600536810019847

**Published:** 2010-05-29

**Authors:** Ivan Potočňák, Zuzana Pravcová, Zdeněk Trávníček

**Affiliations:** a Department of Inorganic Chemistry, Faculty of Science, P.J. Šafárik University, Moyzesova 11, SK-041 54 Košice, Slovakia; b Department of Inorganic Chemistry, Faculty of Science, Palacký University, Tř. 17. listopadu 12, CZ-77146 Olomouc, Czech Republic

## Abstract

In the title compound, [Cu(C_2_N_3_)_2_(C_12_H_6_Cl_2_N_2_)_2_], the Cu^II^ atom is coordinated by two chelating 4,7-dichloro-1,10-phenanthroline (4,7-Cl-phen) ligands and two dicyanamide (dca) ligands in a *cis* arrangement, forming a distorted octa­hedral geometry. The equatorial plane is occupied by three N atoms from two 4,7-Cl-phen ligands and one N atom from a dca ligand at shorter Cu—N distances. Due to the Jahn–Teller effect, the axial positions are occupied by a 4,7-Cl-phen N atom and a dca N atom at longer Cu—N distances. The dca ligands are nearly planar, with a maximum deviations of 0.006 (1) Å. The crystal structure is stabilized by weak C—H⋯N hydrogen bonds, with cyanide N atoms as acceptors, and π–π inter­actions between adjacent phenyl rings [centroid–centroid distance = 3.725 (3) Å].

## Related literature

For long-range magnetic ordering in *M*(dca)_2_ compounds, see: Batten & Murray (2003 ▶); Kurmoo & Kepert (1998 ▶). For penta-coordinated Cu(II) in [Cu(*L*)_2_dca]*Y* complexes [*L* = 1,10-phenanthroline (phen) and 2,2′-bipyridine (bpy), *Y* = a monovalent anion], see: Potočňák *et al.* (2005 ▶, 2008 ▶). For related structures of [*M*(phen)_2_(dca)_2_] compounds, see: Lan *et al.* (2005 ▶) *(M* = Cd); Potočňák *et al.* (1995 ▶) (*M* = Cu); Wang *et al.* (2000 ▶) (*M* = Mn and Zn); Wu *et al.* (2004 ▶) (*M* = Ni). For typical N—C*sp* bond lengths, see: Jolly (1991 ▶). For π–π inter­actions, see: Janiak (2000 ▶).
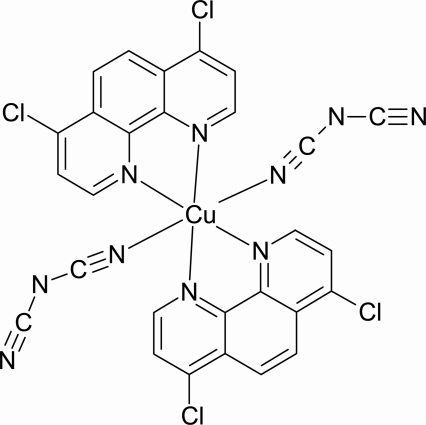


## Experimental

### 

#### Crystal data

[Cu(C_2_N_3_)_2_(C_12_H_6_Cl_2_N_2_)_2_]
*M* *_r_* = 693.82
Monoclinic, 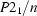

*a* = 9.5484 (2) Å
*b* = 16.6471 (3) Å
*c* = 17.4906 (3) Å
β = 97.316 (2)°
*V* = 2757.55 (9) Å^3^
*Z* = 4
Mo *K*α radiation
μ = 1.22 mm^−1^
*T* = 110 K
0.30 × 0.25 × 0.20 mm

#### Data collection

Oxford Diffraction CCD diffractometer
Absorption correction: multi-scan (*CrysAlis RED*; Oxford Diffraction, 2006 ▶) *T*_min_ = 0.711, *T*_max_ = 0.792
24411 measured reflections
5408 independent reflections
4583 reflections with *I* > 2σ(*I*)
*R*_int_ = 0.018

#### Refinement

*R*[*F* ^2^ > 2σ(*F* ^2^)] = 0.031
*wR*(*F* ^2^) = 0.082
*S* = 1.06
5408 reflections
388 parameters
H-atom parameters constrained
Δρ_max_ = 0.64 e Å^−3^
Δρ_min_ = −0.23 e Å^−3^

### 

Data collection: *CrysAlis CCD* (Oxford Diffraction, 2006 ▶); cell refinement: *CrysAlis RED* (Oxford Diffraction, 2006 ▶); data reduction: *CrysAlis RED*; program(s) used to solve structure: *SHELXS97* (Sheldrick, 2008 ▶); program(s) used to refine structure: *SHELXL97* (Sheldrick, 2008 ▶); molecular graphics: *DIAMOND* (Brandenburg, 1999 ▶); software used to prepare material for publication: *SHELXL97*.

## Supplementary Material

Crystal structure: contains datablocks I, global. DOI: 10.1107/S1600536810019847/hy2310sup1.cif

Structure factors: contains datablocks I. DOI: 10.1107/S1600536810019847/hy2310Isup2.hkl

Additional supplementary materials:  crystallographic information; 3D view; checkCIF report

## Figures and Tables

**Table 1:** Selected bond lengths (Å)

Cu1—N1	1.9707 (19)
Cu1—N40	2.0267 (17)
Cu1—N30	2.0431 (16)
Cu1—N10	2.0575 (17)
Cu1—N4	2.2863 (19)
Cu1—N20	2.3715 (17)

**Table 2:** Hydrogen-bond geometry (Å, °)

*D*—H⋯*A*	*D*—H	H⋯*A*	*D*⋯*A*	*D*—H⋯*A*
C12—H12⋯N4	0.93	2.49	3.143 (3)	127
C22—H22⋯N3^i^	0.93	2.46	3.304 (3)	151
C32—H32⋯N6^ii^	0.93	2.54	3.181 (3)	126
C43—H43⋯N6^iii^	0.93	2.53	3.107 (3)	120

Symmetry codes: (i) 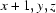; (ii) 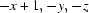; (iii) 
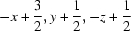
.

**Fig. 1.**
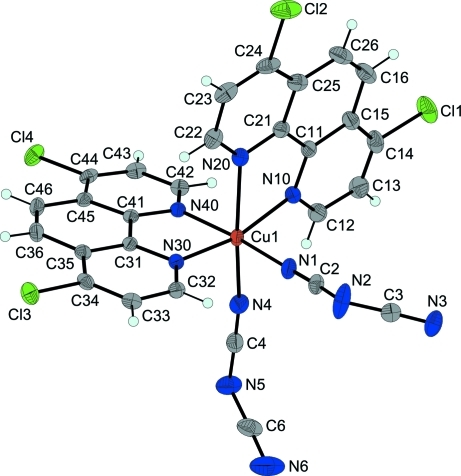
The molecular structure of the title compound, with 50% probability displacement ellipsoids for non-H atoms.

**Fig. 2.**
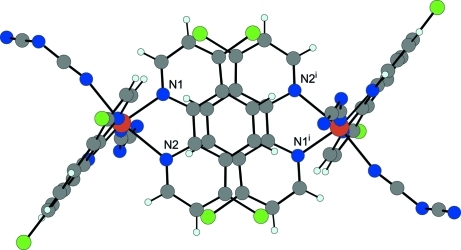
Parallel stacking of phenyl rings enabling π–π interactions in the title compound. [Symmetry code: (i) 1-x, 1-y, -z.]

## supplementary crystallographic information

### Comment

Nowadays, there is increasing interest in the synthesis and characterization of new coordination compounds due to their fascinating structural features. Among the various classes of ligands currently employed for the generation of coordination compounds, dicyanamide (dca) has been attracting a lot of attention, partly due to the discovery of long-range magnetic ordering in the M(dca)_2_ compounds (Batten & Murray, 2003; Kurmoo & Kepert, 1998). A particular feature of this ligand is the variability in coordination modes it can display and thus it is able to generate one- to three-dimensional networks, as well as molecular and ionic compounds, depending on its metallic centers and its organic coligands. In our previous work with pseudohalides we have used dca and nitrosodicyanomethanide within our study on the spectral–structural correlations of penta-coordinated [Cu(L)_2_dca]Y complexes [L = 1,10-phenanthroline (phen) and 2,2'-bipyridine (bpy), Y = a monovalent anion] (Potočňák *et al.*, 2005, 2008). With the aim to continue in this work we used 4,7-dichloro-1,10-phenanthroline (4,7-Cl-phen) in our synthesis and here we present the structure of accidentally prepared the title compound.

The title compound is formed by discrete molecules (Fig. 1) held together by weak hydrogen bonds and π–π interactions. The Cu^II^ atom is coordinated by two chelating 4,7-Cl-phen molecules and by two dicyanamide ligands in a *cis* arrangement, forming a distorted octahedral geometry. Similar *cis* coordination of two dca ligands was observed in [M(phen)_2_(dca)_2_] compounds with M = Ni (Wu *et al.*, 2004), Cd (Lan *et al.*, 2005), Mn and Zn (Wang *et al.*, 2000) and Cu (Potočňák *et al.*, 1995), which are mutually isostructural. The equatorial plane in the title compound is occupied by three N atoms of two 4,7-Cl-phen molecules with Cu—N distances between 2.0267 (17) and 2.0575 (17) Å while the fourth position is occupied by N1 atom of dca at a shorter distance of 1.9707 (18) Å (Table 1). Due to Jahn-Teller effect the axial positions are occupied at longer distances [Cu1—N4 = 2.2863 (19) and Cu1—N20 = 2.3715 (17) Å]. The two dca ligands are perfectly planar, with the largest deviation of atoms from the mean planes being 0.006 (1) Å. All N_cyanide_≡C distances [1.147 (6) Å in average] are usual for triple N≡C bond (1.15 Å) whereas N_amide_—C distances [1.303 (10) Å in average] are slightly shorter than typical N—C_sp_ bond (1.35 Å) (Jolly, 1991). The bond angles around cyanide C atoms are, as expected, nearly linear [175 (2)° in average] and the angles around amide N atoms are consistent with sp^2^ hybridization [121 (5)° in average]. All mentioned values of bonds and angles are close to the values observed in the above mentioned [M(phen)_2_(dca)_2_] compounds. Aromatic rings of two 4,7-Cl-phen molecules are nearly planar; the largest deviation of atoms from their mean planes is 0.095 (1) Å and the bond distances and angles (including Cl atoms) are normal.

The structure of the title compound is stabilized by weak C—H···N hydrogen bonds with cyanide N atoms of the dca ligands as acceptors (Table 2). The next stabilization comes from face to face π–π interactions (Janiak, 2000) between parallel phenyl rings of two adjacent 4,7-Cl-phen molecules (Fig. 2) as evidenced by the distance of Cg(phenyl)···Cg(phenyl)^i^ = 3.725 (3) Å and by the angle between phenyl ring normal and vector connecting Cg and Cg^i^ of 18.5° [symmetry code: (i) = 1-*x*, 1-*y*, -*z*].

### Experimental

The title compound was prepared by chance during our attempts to prepare [Cu(4,7-Cl-phen)_2_(dca)]NO_3_ compound with a penta-coordinated Cu^II^ atom. Crystals of the title compound were prepared by mixing a 0.1 *M* aqueous solution of Cu(NO_3_)_2_ (1 mmol, 10 ml) with a 0.1 *M* ethanolic solution of 4,7-Cl-phen (2 mmol, 20 ml). To the resulting dark green solution, a 0.1 *M* aqueous solution of NaN(CN)_2_ (1 mmol, 10 ml) was added (all solutions were warmed before mixing). After few days, dark green crystals were filtered off and dried in air.

### Refinement

H atom were positioned geometrically and refined as riding atoms, with C—H = 0.93 Å and *U*_iso_(H) = 1.2*U*_eq_(C).

### Crystal data

**Table:**

[Cu(C_2_N_3_)_2_(C_12_H_6_Cl_2_N_2_)_2_]	*F*(000) = 1388
*M**_r_* = 693.82	*D*_x_ = 1.671 Mg m^−^^3^
Monoclinic, *P*2_1_/*n*	Mo *K*α radiation, λ = 0.71073 Å
Hall symbol: -P 2yn	Cell parameters from 18055 reflections
*a* = 9.5484 (2) Å	θ = 2.6–32.0°
*b* = 16.6471 (3) Å	µ = 1.22 mm^−^^1^
*c* = 17.4906 (3) Å	*T* = 110 K
β = 97.316 (2)°	Prism, dark green
*V* = 2757.55 (9) Å^3^	0.30 × 0.25 × 0.20 mm
*Z* = 4	

### Data collection

**Table:**

Oxford Diffraction CCD diffractometer	5408 independent reflections
Radiation source: Enhance Mo X-ray Source	4583 reflections with *I* > 2σ(*I*)
graphite	*R*_int_ = 0.018
Detector resolution: 8.3611 pixels mm^-1^	θ_max_ = 26.0°, θ_min_ = 2.6°
Rotation method data acquisition using ω scans	*h* = −11→11
Absorption correction: multi-scan (*CrysAlis RED*; Oxford Diffraction, 2006)	*k* = −20→17
*T*_min_ = 0.711, *T*_max_ = 0.792	*l* = −20→21
24411 measured reflections	

### Refinement

**Table:**

Refinement on *F*^2^	Primary atom site location: structure-invariant direct methods
Least-squares matrix: full	Secondary atom site location: difference Fourier map
*R*[*F*^2^ > 2σ(*F*^2^)] = 0.031	Hydrogen site location: inferred from neighbouring sites
*wR*(*F*^2^) = 0.082	H-atom parameters constrained
*S* = 1.06	*w* = 1/[σ^2^(*F*_o_^2^) + (0.0472*P*)^2^ + 1.3402*P*] where *P* = (*F*_o_^2^ + 2*F*_c_^2^)/3
5408 reflections	(Δ/σ)_max_ = 0.001
388 parameters	Δρ_max_ = 0.64 e Å^−^^3^
0 restraints	Δρ_min_ = −0.23 e Å^−^^3^

### Fractional atomic coordinates and isotropic or equivalent isotropic displacement parameters (Å^2^)

**Table:**

	*x*	*y*	*z*	*U*_iso_*/*U*_eq_	
Cu1	0.73584 (2)	0.244567 (14)	0.074294 (14)	0.01736 (8)	
N10	0.65586 (17)	0.34366 (10)	0.12412 (10)	0.0201 (4)	
N20	0.81350 (18)	0.35667 (10)	0.00736 (10)	0.0204 (4)	
Cl1	0.44014 (7)	0.55644 (4)	0.21633 (4)	0.04029 (16)	
Cl2	0.85895 (7)	0.58898 (4)	−0.11851 (4)	0.04439 (18)	
C11	0.6665 (2)	0.41712 (12)	0.09164 (11)	0.0190 (4)	
C12	0.5825 (2)	0.33722 (14)	0.18341 (13)	0.0255 (5)	
H12	0.5760	0.2870	0.2061	0.031*	
C13	0.5150 (2)	0.40176 (14)	0.21322 (13)	0.0295 (5)	
H13	0.4644	0.3948	0.2548	0.035*	
C14	0.5242 (2)	0.47530 (14)	0.18044 (13)	0.0277 (5)	
C15	0.6010 (2)	0.48611 (13)	0.11737 (12)	0.0233 (5)	
C16	0.6149 (3)	0.56097 (13)	0.07980 (14)	0.0313 (5)	
H16	0.5720	0.6064	0.0972	0.038*	
C21	0.7488 (2)	0.42388 (12)	0.02814 (11)	0.0199 (4)	
C22	0.8939 (2)	0.36242 (14)	−0.04874 (12)	0.0246 (5)	
H22	0.9409	0.3167	−0.0623	0.030*	
C23	0.9115 (2)	0.43357 (14)	−0.08856 (13)	0.0287 (5)	
H23	0.9685	0.4352	−0.1279	0.034*	
C24	0.8431 (2)	0.50057 (14)	−0.06831 (13)	0.0285 (5)	
C25	0.7597 (2)	0.49893 (13)	−0.00764 (12)	0.0249 (5)	
C26	0.6891 (3)	0.56708 (13)	0.01959 (14)	0.0322 (5)	
H26	0.6946	0.6164	−0.0048	0.039*	
N30	0.84783 (17)	0.16751 (10)	0.01434 (9)	0.0171 (3)	
N40	0.92200 (17)	0.24532 (9)	0.14439 (10)	0.0167 (3)	
Cl3	1.15245 (6)	0.02029 (3)	−0.10381 (3)	0.02774 (13)	
Cl4	1.35419 (5)	0.22801 (4)	0.27827 (3)	0.02713 (13)	
C31	0.9859 (2)	0.16187 (11)	0.04440 (11)	0.0160 (4)	
C32	0.8057 (2)	0.12642 (12)	−0.04913 (11)	0.0195 (4)	
H32	0.7110	0.1289	−0.0696	0.023*	
C33	0.8970 (2)	0.07940 (12)	−0.08686 (12)	0.0211 (4)	
H33	0.8634	0.0508	−0.1311	0.025*	
C34	1.0362 (2)	0.07628 (12)	−0.05764 (12)	0.0199 (4)	
C35	1.0863 (2)	0.11723 (12)	0.01083 (11)	0.0185 (4)	
C36	1.2283 (2)	0.11490 (13)	0.04832 (12)	0.0227 (4)	
H36	1.2964	0.0868	0.0256	0.027*	
C41	1.0252 (2)	0.20322 (11)	0.11568 (11)	0.0154 (4)	
C42	0.9524 (2)	0.27980 (12)	0.21246 (11)	0.0199 (4)	
H42	0.8820	0.3086	0.2325	0.024*	
C43	1.0851 (2)	0.27489 (13)	0.25544 (12)	0.0214 (4)	
H43	1.1023	0.2990	0.3037	0.026*	
C44	1.1897 (2)	0.23404 (12)	0.22564 (12)	0.0197 (4)	
C45	1.1636 (2)	0.19653 (12)	0.15307 (11)	0.0176 (4)	
C46	1.2653 (2)	0.15285 (13)	0.11640 (12)	0.0226 (4)	
H46	1.3584	0.1504	0.1396	0.027*	
C1	0.4712 (2)	0.22929 (13)	−0.04859 (13)	0.0232 (5)	
N1	0.56293 (19)	0.23404 (11)	0.00040 (11)	0.0258 (4)	
N2	0.3809 (2)	0.22103 (17)	−0.10946 (12)	0.0451 (6)	
C3	0.2472 (2)	0.23853 (13)	−0.11338 (12)	0.0233 (5)	
N3	0.1290 (2)	0.25177 (13)	−0.12394 (12)	0.0348 (5)	
C4	0.6328 (2)	0.10062 (14)	0.19498 (13)	0.0251 (5)	
N4	0.6632 (2)	0.15641 (12)	0.16144 (11)	0.0299 (4)	
N5	0.6030 (2)	0.03864 (12)	0.23565 (12)	0.0361 (5)	
C6	0.4981 (3)	−0.00718 (14)	0.20559 (14)	0.0352 (6)	
N6	0.4072 (3)	−0.04946 (14)	0.18408 (16)	0.0607 (8)	

### Atomic displacement parameters (Å^2^)

**Table:**

	*U*^11^	*U*^22^	*U*^33^	*U*^12^	*U*^13^	*U*^23^
Cu1	0.01357 (13)	0.01900 (14)	0.01928 (14)	0.00215 (9)	0.00121 (10)	−0.00168 (10)
N10	0.0195 (9)	0.0188 (9)	0.0220 (9)	0.0018 (7)	0.0030 (7)	0.0003 (7)
N20	0.0201 (9)	0.0234 (9)	0.0171 (9)	0.0008 (7)	0.0004 (7)	0.0003 (7)
Cl1	0.0458 (4)	0.0343 (3)	0.0409 (4)	0.0157 (3)	0.0060 (3)	−0.0142 (3)
Cl2	0.0578 (4)	0.0363 (3)	0.0376 (3)	−0.0181 (3)	0.0007 (3)	0.0157 (3)
C11	0.0166 (10)	0.0195 (10)	0.0195 (10)	0.0006 (8)	−0.0028 (8)	−0.0009 (8)
C12	0.0264 (11)	0.0251 (11)	0.0266 (12)	0.0005 (9)	0.0100 (9)	0.0005 (9)
C13	0.0296 (12)	0.0324 (13)	0.0286 (12)	0.0011 (10)	0.0114 (10)	−0.0058 (10)
C14	0.0256 (11)	0.0289 (12)	0.0278 (12)	0.0080 (9)	0.0002 (9)	−0.0107 (10)
C15	0.0235 (11)	0.0207 (11)	0.0239 (11)	0.0027 (9)	−0.0041 (9)	−0.0045 (9)
C16	0.0376 (13)	0.0198 (11)	0.0343 (13)	0.0059 (10)	−0.0042 (11)	−0.0044 (10)
C21	0.0178 (10)	0.0213 (10)	0.0190 (10)	−0.0014 (8)	−0.0037 (8)	0.0003 (8)
C22	0.0219 (11)	0.0333 (12)	0.0179 (10)	−0.0009 (9)	−0.0006 (9)	0.0001 (9)
C23	0.0269 (12)	0.0404 (14)	0.0183 (11)	−0.0100 (10)	0.0014 (9)	0.0032 (10)
C24	0.0317 (12)	0.0290 (12)	0.0228 (11)	−0.0126 (10)	−0.0049 (9)	0.0078 (9)
C25	0.0266 (11)	0.0231 (11)	0.0223 (11)	−0.0042 (9)	−0.0068 (9)	0.0026 (9)
C26	0.0421 (14)	0.0187 (11)	0.0330 (13)	−0.0019 (10)	−0.0056 (11)	0.0040 (10)
N30	0.0170 (8)	0.0166 (8)	0.0171 (8)	0.0007 (7)	0.0006 (7)	0.0019 (7)
N40	0.0173 (8)	0.0164 (8)	0.0165 (8)	−0.0003 (6)	0.0030 (7)	0.0023 (7)
Cl3	0.0304 (3)	0.0281 (3)	0.0261 (3)	0.0076 (2)	0.0089 (2)	−0.0045 (2)
Cl4	0.0191 (3)	0.0393 (3)	0.0212 (3)	−0.0046 (2)	−0.0043 (2)	0.0036 (2)
C31	0.0159 (9)	0.0151 (10)	0.0168 (10)	−0.0004 (8)	0.0017 (8)	0.0038 (8)
C32	0.0192 (10)	0.0198 (10)	0.0189 (10)	−0.0010 (8)	0.0002 (8)	0.0017 (8)
C33	0.0280 (11)	0.0185 (10)	0.0166 (10)	−0.0023 (8)	0.0024 (8)	0.0002 (8)
C34	0.0247 (11)	0.0161 (10)	0.0203 (10)	0.0031 (8)	0.0079 (8)	0.0037 (8)
C35	0.0203 (10)	0.0157 (10)	0.0197 (10)	0.0001 (8)	0.0038 (8)	0.0034 (8)
C36	0.0184 (10)	0.0233 (11)	0.0269 (11)	0.0053 (8)	0.0050 (9)	0.0021 (9)
C41	0.0166 (9)	0.0144 (9)	0.0154 (10)	−0.0012 (7)	0.0033 (8)	0.0043 (7)
C42	0.0232 (10)	0.0195 (10)	0.0173 (10)	−0.0015 (8)	0.0041 (8)	−0.0010 (8)
C43	0.0258 (11)	0.0225 (10)	0.0157 (10)	−0.0057 (9)	0.0013 (8)	0.0012 (8)
C44	0.0171 (10)	0.0220 (10)	0.0189 (10)	−0.0063 (8)	−0.0015 (8)	0.0062 (8)
C45	0.0167 (9)	0.0169 (10)	0.0189 (10)	−0.0022 (8)	0.0016 (8)	0.0049 (8)
C46	0.0148 (10)	0.0257 (11)	0.0265 (11)	0.0027 (8)	−0.0002 (8)	0.0052 (9)
C1	0.0180 (10)	0.0276 (11)	0.0252 (11)	0.0034 (9)	0.0071 (9)	0.0000 (9)
N1	0.0165 (9)	0.0299 (10)	0.0302 (10)	0.0035 (7)	0.0008 (8)	−0.0027 (8)
N2	0.0204 (10)	0.0888 (18)	0.0253 (11)	0.0133 (11)	−0.0003 (8)	−0.0157 (11)
C3	0.0269 (12)	0.0259 (11)	0.0168 (11)	−0.0018 (9)	0.0010 (9)	0.0001 (8)
N3	0.0189 (11)	0.0511 (14)	0.0326 (11)	0.0036 (9)	−0.0041 (8)	0.0005 (9)
C4	0.0213 (11)	0.0269 (12)	0.0270 (12)	0.0033 (9)	0.0023 (9)	−0.0039 (10)
N4	0.0246 (10)	0.0295 (11)	0.0366 (11)	−0.0012 (8)	0.0081 (8)	0.0041 (9)
N5	0.0401 (12)	0.0328 (11)	0.0325 (11)	−0.0109 (9)	−0.0071 (9)	0.0076 (9)
C6	0.0446 (15)	0.0224 (12)	0.0347 (14)	−0.0047 (11)	−0.0094 (11)	0.0068 (10)
N6	0.0718 (18)	0.0376 (14)	0.0632 (17)	−0.0228 (13)	−0.0276 (14)	0.0153 (12)

### Geometric parameters (Å, °)

**Table:**

Cu1—N1	1.9707 (19)	N30—C31	1.359 (2)
Cu1—N40	2.0267 (17)	N40—C42	1.320 (3)
Cu1—N30	2.0431 (16)	N40—C41	1.357 (2)
Cu1—N10	2.0575 (17)	Cl3—C34	1.727 (2)
Cu1—N4	2.2863 (19)	Cl4—C44	1.719 (2)
Cu1—N20	2.3715 (17)	C31—C35	1.400 (3)
N10—C12	1.328 (3)	C31—C41	1.432 (3)
N10—C11	1.358 (3)	C32—C33	1.398 (3)
N20—C22	1.324 (3)	C32—H32	0.9300
N20—C21	1.350 (3)	C33—C34	1.363 (3)
Cl1—C14	1.729 (2)	C33—H33	0.9300
Cl2—C24	1.730 (2)	C34—C35	1.408 (3)
C11—C15	1.408 (3)	C35—C36	1.429 (3)
C11—C21	1.445 (3)	C36—C46	1.354 (3)
C12—C13	1.388 (3)	C36—H36	0.9300
C12—H12	0.9300	C41—C45	1.402 (3)
C13—C14	1.359 (3)	C42—C43	1.391 (3)
C13—H13	0.9300	C42—H42	0.9300
C14—C15	1.412 (3)	C43—C44	1.366 (3)
C15—C16	1.423 (3)	C43—H43	0.9300
C16—C26	1.346 (4)	C44—C45	1.408 (3)
C16—H16	0.9300	C45—C46	1.428 (3)
C21—C25	1.407 (3)	C46—H46	0.9300
C22—C23	1.395 (3)	C1—N1	1.147 (3)
C22—H22	0.9300	C1—N2	1.289 (3)
C23—C24	1.362 (3)	N2—C3	1.302 (3)
C23—H23	0.9300	C3—N3	1.142 (3)
C24—C25	1.406 (3)	C4—N4	1.155 (3)
C25—C26	1.432 (3)	C4—N5	1.305 (3)
C26—H26	0.9300	N5—C6	1.314 (3)
N30—C32	1.322 (3)	C6—N6	1.143 (3)
			
N1—Cu1—N40	173.85 (7)	C16—C26—C25	121.2 (2)
N1—Cu1—N30	93.27 (7)	C16—C26—H26	119.4
N40—Cu1—N30	80.69 (6)	C25—C26—H26	119.4
N1—Cu1—N10	91.37 (7)	C32—N30—C31	117.75 (17)
N40—Cu1—N10	94.76 (6)	C32—N30—Cu1	129.23 (13)
N30—Cu1—N10	165.36 (7)	C31—N30—Cu1	112.97 (13)
N1—Cu1—N4	94.60 (7)	C42—N40—C41	118.15 (17)
N40—Cu1—N4	85.29 (7)	C42—N40—Cu1	128.54 (14)
N30—Cu1—N4	99.33 (7)	C41—N40—Cu1	113.30 (13)
N10—Cu1—N4	94.12 (7)	N30—C31—C35	123.84 (18)
N1—Cu1—N20	91.99 (7)	N30—C31—C41	115.94 (17)
N40—Cu1—N20	89.34 (6)	C35—C31—C41	120.20 (17)
N30—Cu1—N20	91.36 (6)	N30—C32—C33	122.97 (19)
N10—Cu1—N20	74.60 (6)	N30—C32—H32	118.5
N4—Cu1—N20	167.08 (6)	C33—C32—H32	118.5
C12—N10—C11	118.32 (18)	C34—C33—C32	118.78 (19)
C12—N10—Cu1	121.83 (14)	C34—C33—H33	120.6
C11—N10—Cu1	119.57 (14)	C32—C33—H33	120.6
C22—N20—C21	117.89 (18)	C33—C34—C35	120.69 (18)
C22—N20—Cu1	132.06 (15)	C33—C34—Cl3	119.97 (16)
C21—N20—Cu1	109.68 (13)	C35—C34—Cl3	119.33 (15)
N10—C11—C15	122.76 (19)	C31—C35—C34	115.91 (18)
N10—C11—C21	117.96 (18)	C31—C35—C36	118.81 (18)
C15—C11—C21	119.28 (18)	C34—C35—C36	125.26 (18)
N10—C12—C13	123.2 (2)	C46—C36—C35	121.13 (19)
N10—C12—H12	118.4	C46—C36—H36	119.4
C13—C12—H12	118.4	C35—C36—H36	119.4
C14—C13—C12	118.7 (2)	N40—C41—C45	123.67 (18)
C14—C13—H13	120.6	N40—C41—C31	116.63 (17)
C12—C13—H13	120.6	C45—C41—C31	119.66 (17)
C13—C14—C15	120.8 (2)	N40—C42—C43	122.83 (19)
C13—C14—Cl1	119.50 (18)	N40—C42—H42	118.6
C15—C14—Cl1	119.69 (18)	C43—C42—H42	118.6
C11—C15—C14	116.19 (19)	C44—C43—C42	118.91 (19)
C11—C15—C16	119.7 (2)	C44—C43—H43	120.5
C14—C15—C16	124.1 (2)	C42—C43—H43	120.5
C26—C16—C15	121.1 (2)	C43—C44—C45	120.73 (19)
C26—C16—H16	119.4	C43—C44—Cl4	119.18 (16)
C15—C16—H16	119.4	C45—C44—Cl4	120.10 (16)
N20—C21—C25	123.64 (19)	C41—C45—C44	115.67 (18)
N20—C21—C11	117.06 (18)	C41—C45—C46	119.00 (18)
C25—C21—C11	119.29 (19)	C44—C45—C46	125.33 (18)
N20—C22—C23	123.4 (2)	C36—C46—C45	121.06 (18)
N20—C22—H22	118.3	C36—C46—H46	119.5
C23—C22—H22	118.3	C45—C46—H46	119.5
C24—C23—C22	118.2 (2)	N1—C1—N2	172.2 (2)
C24—C23—H23	120.9	C1—N1—Cu1	172.76 (18)
C22—C23—H23	120.9	C1—N2—C3	124.6 (2)
C23—C24—C25	121.1 (2)	N3—C3—N2	173.5 (2)
C23—C24—Cl2	119.25 (18)	N4—C4—N5	177.2 (2)
C25—C24—Cl2	119.61 (18)	C4—N4—Cu1	166.37 (18)
C24—C25—C21	115.7 (2)	C4—N5—C6	116.7 (2)
C24—C25—C26	124.9 (2)	N6—C6—N5	175.5 (3)
C21—C25—C26	119.4 (2)		

### Hydrogen-bond geometry (Å, °)

**Table:**

*D*—H···*A*	*D*—H	H···*A*	*D*···*A*	*D*—H···*A*
C12—H12···N4	0.93	2.49	3.143 (3)	127
C22—H22···N3^i^	0.93	2.46	3.304 (3)	151
C32—H32···N6^ii^	0.93	2.54	3.181 (3)	126
C43—H43···N6^iii^	0.93	2.53	3.107 (3)	120

Symmetry codes: (i) *x*+1, *y*, *z*; (ii) −*x*+1, −*y*, −*z*; (iii) −*x*+3/2, *y*+1/2, −*z*+1/2.
